# Fostering hope and recovery: enhancing psychological resources in military personnel with post-traumatic stress disorder

**DOI:** 10.1186/s40779-025-00604-4

**Published:** 2025-04-09

**Authors:** Bénédicte Aubet, Charles Martin-Krumm, Marion Trousselard

**Affiliations:** 1 École de Psychologues Praticiens (EPP), 23 rue du Montparnasse, 75006 Paris, France; 2 Stress Neurophysiology Unit, Department of Neurosciences and Cognitive Sciences, Institut de Recherche Biomédicale des Armées (IRBA), BP 73, 91223 Brétigny-Sur-Orge Cedex, France; 3 UMR 1319 INSPIIRE Inserm, Lorraine University, 57000 Metz, France; 4 École du Val de Grâce, 1 Place Laveran, 75005 Paris, France

**Keywords:** Post-traumatic stress disorder, Hope, Self-esteem, Positive mental health, Well-being, Military personnel, Rehabilitation

 Dear Editor,

Post-traumatic stress disorder (PTSD) is a major issue for military personnel, with prevalence rates between 1 and 35% in veterans [1], significantly higher than in the general population [2]. Psychological resources, particularly hope, can protect against PTSD and promote post-traumatic growth [3]. Hope, conceptualized as both a trait and a state, contributes to well-being and resilience and is negatively associated with PTSD symptoms, representing a psychological factor while mitigating the impact of trauma by fostering resilience and adaptive coping mechanisms. Thus, individuals with higher levels of hope tend to exhibit lower levels of PTSD symptoms [4, 5]. The Adult Dispositional Hope Scale (ADHS) measures 2 main components of hope: agency (goal-directed energy) and pathways (planning to accomplish goals), providing a comprehensive assessment of an individual’s hope levels [5]. Empirical evidence supports the effectiveness of hope-based interventions in reducing PTSD symptoms and improving psychological functioning. Studies have shown that programs incorporating elements of hope theory can significantly enhance resilience and foster post-traumatic growth [6, 7]. Encouraging individuals to set meaningful and achievable goals provides direction and purpose, which are essential for trauma recovery. Fostering a sense of agency, or the belief in one’s ability to influence outcomes, empowers individuals to take proactive steps in their recovery journey. Additionally, promoting pathways thinking, or developing multiple strategies to achieve goals, helps individuals overcome obstacles and fosters a sense of control over their circumstances. These mechanisms work together to counteract feelings of helplessness and hopelessness commonly observed in individuals with PTSD. Increasing hope offers several clinical benefits for individuals with PTSD. Hopeful individuals are more likely to engage with challenges rather than avoid them, which can reduce avoidance symptoms, a hallmark feature of PTSD. Higher levels of hope are also associated with improved coping strategies, such as problem-solving and seeking social support, as well as enhanced emotional regulation by providing a framework for understanding and processing traumatic experiences. Furthermore, hope has been shown to positively influence psychological resources such as self-esteem, positive mental health (PMH), and well-being [6, 7]. These resources are vital for recovery because they enable individuals to reframe their experiences, build resilience, and sustain motivation during the healing process. For example, higher self-esteem can reinforce a sense of agency, while improved well-being promotes adaptive coping mechanisms that align with hope-based interventions [4].

Building on this theoretical foundation, this study aimed to evaluate the impact of the Centre de Ressources des Blessés de l’Armée de Terre (CREBAT) program on psychological resources in military personnel with PTSD. Specifically, we sought to determine whether: 1) the CREBAT intervention enhances self-esteem, PMH, and well-being; and 2) whether PTSD severity and hope levels influence these psychological resources. The CREBAT is a French Army initiative designed to support soldiers recovering from psychological trauma, including PTSD. This 1-week program provides tailored interventions aimed at enhancing resilience and psychological recovery through structured activities such as group workshops, physical challenges, and reflective exercises. These activities are designed to promote self-confidence, emotional regulation, and social reintegration, key elements for fostering hope while addressing PTSD symptoms.

We hypothesized that the CREBAT intervention would significantly impact resource growth, and that PTSD severity and hope levels would have a significant effect on the assessed resources. Forty-two veterans (41 men, 1 woman; age range 22.0–51.0 years) participating in the 1-week CREBAT reinsertion course were recruited. Participants completed the Post-Traumatic Stress Disorder Checklist for DSM-5 (PCL-5), ADHS, Rosenberg Self-Esteem Scale (RSES), Mental Health Continuum-Short Form (MHC-SF), and Warwick–Edinburgh Mental Well-Being Scale (WEMWBS). The RSES, MHC-SF, and WEMWBS were administered before and after the CREBAT course, while the PCL-5 and ADHS were only administered before. Detailed methods are provided in Additional file 1: Methods. The general characteristics of the samples are presented in the Additional file 1: Table S1.

Results showed that the CREBAT intervention significantly improved all 3 assessed psychological resources. Self-esteem increased [*t*(41,42) = −2.07, *P*_0.02_ < 0.05, Cohen’s* d* = −0.32], with mean scores rising from 26.29 to 27.60 (M_DELTA_ = 1.31); PMH showed substantial improvement [*t*(41,42) = −5.16, *P* < 0.001, Cohen’s *d* = −0.80], with mean scores increasing from 25.14 to 34.60 (M_DELTA_ = 8.02); well-being significantly enhanced (*W* = 91.50, *P* < 0.001, Hodges-Lehmann estimate = −8.00, rank-biserial correlation = −0.79), with mean scores rising from 40.83 to 48.86 (M_DELTA_ = 8.02) (Additional file 1: Table S2). These results demonstrate CREBAT’s effectiveness in improving key psychological resources in veterans with PTSD, with the strongest effect on PMH, followed by well-being, and a smaller but significant impact on self-esteem. To further understand the relationship between PTSD, hope, and resource recovery, we conducted a *k*-means clustering analysis based on participants’ PCL-5 and ADHS scores. This analysis revealed 3 distinct profiles: vulnerabilities (*n* = 19, high PTSD, low hope), resources (*n* = 10, low PTSD, high hope), and mixed (*n* = 13, intermediate levels) (Additional file 1: Table S3).

ANOVAs showed significant profile effects on self-esteem [*F*(2,42) = 8.08, *P* = 0.003, η^2^ = 0.29], with differences between vulnerabilities (M = 3.32) and resources (M = −2.40) (*P* = 0.003) and a trend between vulnerabilities and mixed (M = 0.46) (*P* < 0.10). A marginally significant effect was observed for PMH [*F*(2,42) = 2.40, *P* < 0.10, η^2^ = 0.11], with a trend between vulnerabilities (M = 12.47) and resources (M = 2.20) profiles (*P* < 0.10). No significant differences were found for well-being [*F*(2,42) = 0.10, *P* = 0.38, η^2^ = 0.05]. These results highlight the complex interplay between PTSD severity, hope levels, and the recovery of psychological resources. Notably, individuals in the vulnerabilities profile showed the greatest improvements in self-esteem and PMH, suggesting that those with higher PTSD severity and lower hope may benefit most from the CREBAT intervention in terms of these specific resources. Interestingly, despite the differences in resource recovery, no significant differences in reintegration levels were observed between the profiles (*χ*^2^ = 0*.*82, *P* > 0.05). This suggests that while CREBAT intervention effectively improves psychological resources, the relationship between these improvements and successful reintegration may be more complex and warrants further investigation.

The findings of this study provide valuable insights into the dynamics of psychological resource recovery in veterans with PTSD. The CREBAT intervention demonstrated significant positive impacts on self-esteem, PMH, and well-being, underscoring its effectiveness as a rehabilitation program. The identification of distinct profiles based on PTSD and hope levels offers a nuanced understanding of how these factors influence resource recovery. The differential effects observed across profiles suggest that tailoring interventions to individual characteristics may enhance their effectiveness. For instance, individuals with higher PTSD severity and lower hope levels (the vulnerabilities profile) showed the most substantial gains in self-esteem and PMH, indicating that they may particularly benefit from targeted interventions focusing on these resources. The lack of significant differences in reintegration outcomes between profiles, despite variations in resource recovery, highlights the complexity of the rehabilitation process. This finding suggests that while improving psychological resources is crucial, additional factors may influence successful reintegration into civilian life.

This study’s limitations include small sample size, reliance on self-report measures, gender imbalance with a predominantly male sample, and the absence of a follow-up PTSD assessment; future research should address these issues by replicating the study with a larger, more gender-diverse cohort, incorporating behavioral and physiological indicators, and including a second-phase PCL-5 administration to enhance generalizability, provide more comprehensive insights, investigate potential gender differences, and directly examine changes in PTSD severity. Future studies should address these limitations and explore the long-term impacts of resource recovery on reintegration outcomes. Additionally, investigating the role of other psychological resources and environmental factors in the rehabilitation process could provide a more comprehensive understanding of veteran reintegration.

In conclusion, this study demonstrates that CREBAT intervention significantly impacts the reappropriation of self-esteem, PMH, and well-being in veterans with PTSD. The levels of PTSD and hope influence the recovery of these resources, suggesting the importance of considering existing psychological characteristics in rehabilitation programs. These findings suggest that future rehabilitation programs for PTSD-diagnosed veterans should target hope-related assets, tailor interventions based on individual PTSD and hope levels and investigate additional factors influencing reintegration beyond psychological resource improvement. By focusing on the relationship between PTSD, hope, and psychological resource recovery, this research contributes to the development of more effective, personalized interventions for veterans, potentially enhancing their psychosocial and professional rehabilitation outcomes.

## Supplementary Information

**Additional file 1**: **Methods**. **Table S1** Descriptive data of the sample: biographical characteristics of the participants. **Table S2** General characteristics and statistical normality of the assessed variables. **Table S3** General characteristics of the variables by profile.

## Data Availability

The data that support the findings of this study are available from the corresponding author (Bénédicte Aubet), upon reasonable request.

## Abbreviations

ADHS: Adult dispositional hope scale
CREBAT: Centre de Ressources des Blessés de l’Armée de Terre
MHC-SF: Mental health continuum-short form
PCL-5: Post-traumatic stress disorder checklist for DSM-5
PMH: Positive mental health
PTSD: Post-traumatic stress disorder
RSES: Rosenberg self-esteem scale
WEMWBS: Warwick–Edinburgh mental well-being scale
