# Supplementary material for: Fostering hope and recovery: enhancing psychological resources in military personnel with post-traumatic stress disorder
Source: Mil Med Res. 2025 Apr 9;12:17. doi: 10.1186/s40779-025-00604-4 (PMC11980253; doi:10.1186/s40779-025-00604-4)
Supplement: Supplementary file 1 — Additional file 1: Methods. Table S1 Descriptive data of the sample: biographical characteristics of the participants. Table S2 General characteristics and statistical normality of the assessed variables. Table S3 General characteristics of the variables by profile. [file 40779_2025_604_MOESM1_ESM.doc]

**Methods**

**Procedure**

This follow-up study took place in 2017 and 2021. The project has been approved by the ethics committee of the French Armed Forces Health Service. All 42 subjects received information on the protocol and gave their written consent prior to their participation. Data collection took place in two phases, before and after the rehabilitation and reintegration courses organized by the Centre de Ressources des Blessés de l’Armée de Terre (CREBAT) in 2017 and data analysis and processing in 2021. All 5 scales were administered in French. Three of them were administered twice, once at the beginning of CREBAT and once at the end of CREBAT. Two of them were administered only prior to the CREBAT course. The scales were administered in the same order to the 42 participants in order to ensure the same fatigue effect for all. Finally, the quality of the reintegration of veterans was examined 1 year after the CREBAT intervention.

**Variables**

The 5 endogenous variables [i.e., post-traumatic stress disorder (PTSD), hope, self-esteem (SE), positive mental health (PMH), and well-being (WB)] were operationalized by summing up the results obtained on the items of their respective scales. In the case of hope, the sub-totals of its scale corresponding to sub-dimensions of the resources it evaluates were also deployed in our measures (i.e., pathways and agency factors). The internal consistency of the scales is reported with Cronbach’s alpha [1] and omega coefficient [2]. The psychometric qualities of the French-version scales are reported.

**PTSD**

To investigate the severity of PTSD in the participants, the French version of the four-factor Post-Traumatic Stress Disorder Checklist for DSM-5 (PCL-5) model was used. It is a 20-item self-report scale used to investigate the severity of PTSD in an individual based on the DSM-5 criteria for PTSD symptomatology, which is constructed on a 5-point Likert score of 0 to 4 (i.e., 0 = not at all; 1 = a little bit; 2 = moderately; 3 = quite a bit; 4 = extremely). It was administered only prior to the CREBAT course. The scale shows sound construct validity [*χ*^2^ = 106.16; *df* = 9; *P* < 0.05; comparative fit index (CFI) ≥ 0.95; root mean square error of approximation (RMSEA) ≤ 0.06; standardized root mean square (SRMR) ≤ 0.08] [3]. In this study, the internal consistency of the scale as a whole was satisfactory (α = 0.93 and ω = 0.94).

**Hope agency and hope pathways (HA and HP)**

The examination of hope was done with the French version of the Adult Dispositional Hope Scale (ADHS), which is a 12-item self-administered questionnaire used to assess the level of dispositional hope in an individual. It is based on a 8-point Likert score of 1 to 8 (1 = always false; 2 = false, most of the time; 3 = rather false; 4 = sometimes false; 5 = sometimes true; 6 = rather true; 7 = true, most of the time; 8 = always true). It was administered only prior to the CREBAT course. The scale shows sound construct validity (*χ*^2^ = 30.40; *df* = 19; *P* = 0.047; CFI = 0.91; RMSEA = 0.044; SRMR = 0.050) [4]. In this study, the internal consistency of the scale as a whole was satisfactory (α = 0.80 and ω = 0.81). The agency sub-factor was satisfactory (α = 0.74 and ω = 0.74), as was the pathways sub-factor (α = 0.69 and ω = 0.70).

**SE**

For investigating SE, the French version of the self-reported Rosenberg Self-Esteem Scale (RSES) was administered. It is made up of 10 items validated on a Likert score of 1 to 4 (i.e., 1 = strongly disagree; 2 = somewhat disagree; 3 = somewhat agree; 4 = strongly agree) allowing the evaluation of the global SE that a person may have of himself or herself. The scale shows sound construct validity [*χ*^2^ = 80.22; *df* = 32; *P* < 0.0001; adjusted goodness of fit index (AGFI) = 0.91; SRMR = 0.03] [5]. In this study, the internal consistency of the scale as a whole was very satisfactory in both phases 1 (α = 0.80 and ω = 0.82) and 2 (α = 0.83 and ω = 0.84).

**PMH**

PMH was explored through the Mental Health Continuum-Short Form (MHC-SF), which assesses the individual’s level of PMH through 14 items administered by hetero-assessment and constructed on a six-point Likert score, which ranges from 1 = never to 6 = every day. The scale shows sound construct validity [Satorra-Bentler (SB) *χ*^2^ = 527.96; *df* = 74; *P* < 0.05; CFI = 0.94; RMSEA = 0.07; SRMR = 0.05; Tucker-Lewis index (TLI) = 0.93] [6]. In this study, the internal consistency of the scale as a whole was satisfactory in both phases 1 (α = 0.91 and ω = 0.92) and 2 (α = 0.93 and ω = 0.94).

**WB**

The Warwick-Edinburgh Mental Well-Being Scale (WEMWBS) was administered for the evaluation of WB. It consists of a 14-item scale constructed on a Likert scale of 1 to 5 (i.e., 1 = never; 2 = rarely; 3 = sometimes; 4 = often; 5 = always). The scale shows sound construct validity (*χ*^2^ = 274.21; *df* = 75; *P* < 0.001; CFI = 0.92; RMSEA = 0.07; SRMR = 0.04) [7]. In this study, the internal consistency of the scale as a whole was satisfactory in both phases 1 (α = 0.91 and ω = 0.92) and 2 (α = 0.93 and ω = 0.93).

**Reintegration**

Finally, the level of reintegration of the veterans was assessed according to two categories. Category 0 corresponds to no reintegration and possibly to a loss of contact with the veteran at the conclusion of the CREBAT course and category 1 matches either an ongoing rehabilitation process or an accomplished reintegration into civilian life.

**Data analysis**

Data analyses were conducted using two different tools. Hypotheses were tested in JASP version 0.14.1 statistical software and in Python version 3.10.0 programming language. Data processing and analysis were performed based on the alpha threshold value of ⍺ = 0.05. Therefore, significance was determined at ⍺ < 0.05, trends at (0.05 < ⍺ < 0.10) and non-significance at ⍺ > 0.10. Moreover, results have been rounded to the second decimal place. To test the first hypothesis, that the CREBAT course has an effect on the growth of the studied resources, Student’s *t-*tests were performed to compare the means of SE and PMH recorded before the CREBAT course with those recorded at the end of the CREBAT course, as the Shapiro-Wilk normality tests were not significant (*P* > 0.05), and a Wilcoxon rank-sum test was performed to compare the mean of WB recorded before the CREBAT course with those recorded at the end of the CREBAT course, as the Shapiro-Wilk normality test was significant (*P* < 0.05). The second assumption, which states that distinctive profiles can be identified within our sample based on the severity of PTSD and the level of hope of participants, was then addressed for studying the impact of the profiles on the psychological resources’ recovery. Based on the PCL-5 and ADHS scores, clusters were established among the participants. The general characteristics of the three profiles were then presented, including means, medians, and standard deviations. Then, deltas of resource scores (i.e., post minus before) were calculated for SE, PMH, and WB. Analyses of variances (ANOVAs) were applied to determine whether the profiles behave differently towards deltas of the studied resources as the conditions for normality and homoscedasticity were normal in all three cases (Levene’s *P* > 0.5). Pairwise comparisons provided by the Games-Howell test were used. Finally, the frequencies of reintegration at levels 0 and 1 were computed and modeled for each profile using a *χ*² test.

**References**

1 Cronbach LJ. Coefficient alpha and the internal structure of tests. Psychometrika 1951;16(3):297-334.

2 McDonald RP. Test theory: a unified treatment. Mahwah, NJ: Lawrence Erlbaum; 1999.

3 Ashbaugh AR, Houle-Johnson S, Herbert C, El-Hage W, Brunet A. Psychometric validation of the english and french versions of the Posttraumatic Stress Disorder Checklist for DSM-5 (PCL-5). PLoS One. 2016;11(10):e0161645.

4 Gana K, Daigre S, Ledrich J. Psychometric Properties of the French Version of the Adult Dispositional Hope Scale. Assessment 2013;20(1):114-8.

5 Vallieres EF, Vallerand RJ. Traduction et Validation Canadienne-Française de L’échelle de L’estime de Soi de Rosenberg. Int J Psychol. 1990;25(2):305-16.

6 Doré I, O’Loughlin JL, Sabiston CM, Fournier L. Psychometric evaluation of the Mental Health Continuum-Short Form in French Canadian young adults. Can J Psychiatry. 2017;62(4):286-94.

7 Trousselard M, Steiler D, Dutheil F, Claverie D, Canini F, Fenouillet F, et al. Validation of the Warwick-Edinburgh Mental Well-Being Scale (WEMWBS) in French psychiatric and general populations. Psychiatry Res. 2016;245:282-90.

**Table S1** Descriptive data of the sample: biographical characteristics of the participants (*n* = 42)

| **Item** | **Characteristics** |
| --- | --- |
| Gender [*n*(%)] |  |
| Male | 41 (97.6) |
| Female | 1 (2.4) |
| Marital status [*n*(%)] |  |
| Relationship | 25 (59.5) |
| Single | 17 (40.5) |
| Age (year, mean ± SD) | 33.6 ± 8.2 |
| Age (year, range) | 22.0 – 51.0 |
| DE [*n*(%)] |  |
| Presence | 41 (97.6) |
| Absence | 1 (2.4) |
| DE (mean ± SD) | 4.41 ± 2.04 |
| DE (range) | 0 – 9.00 |
| RDE [*n*(%)] |  |
| Presence | 13 (31.0) |
| Absence | 29 (69.0) |
| RDE (mean ± SD) | 0.50 ± 0.86 |
| RDE (range) | 0 – 3.00 |

*DE* distressing events, *RDE* recent distressing events

**Table S2 General characteristics and statistical normality of the assessed variables (*n* = 42)**

| **Characteristics** | **PTSD** | **HA** | **HP** | **SE** | **PMH** | **WB** |
| --- | --- | --- | --- | --- | --- | --- |
| M_1_ | 45.26 | 19.57 | 22.71 | 26.29 | 25.14 | 40.83 |
| Mean ± SD | 50.00 ± 16.71 | 18.00 ± 5.08 | 23.00 ± 4.74 | 25.50 ± 4.77 | 25.00 ± 13.20 | 40.00 ± 9.63 |
| *P*-value | 0.08 | 0.16 | 0.36 | 0.16 | 0.12 | 0.15 |
| M_2_ |  |  |  | 27.60 | 34.60 | 48.86 |
| Mean ± SD |  |  |  | 27.00 ± 4.63 | 32.50 ± 12.12 | 46.50 ± 8.88 |
| *P*-value |  |  |  | 0.23 | 0.07 | 0.03 |

Variables names are summarized in acronyms where post-traumatic stress disorder (PTSD) severity, hope agency (HA), hope pathways (HP), self-esteem (SE), positive mental health (PMH), well-being (WB); *P*-value is Shapiro-Wilk’s statistical normality test; M_1_ refers to the means of the variables prior to the CREBAT course (phase 1); M_2_ refers to the means of the variables after the CREBAT course (phase 2)

**Table S3 General characteristics of the variables by profile (*n* = 42)**

| **Features** | **PTSD_1_** | **HA_1_** | **HP_1_** | **SE_DELTA_** | **PMH_DELTA_** | **WB_DELTA_** |
| --- | --- | --- | --- | --- | --- | --- |
| Vulnerabilities (*n* = 19) | | | | | | |
| M_1_ or M_DELTA_ | 50.90 | 15.47 | 18.90 | 3.32 | 12.47 | 9.63 |
| Mean ± SD | 52.00 ± 12.98 | 16.00 ± 3.24 | 20.00 ± 3.71 | 4.00 ± 3.80 | 7.00 ± 13.82 | 7.00 ± 10.46 |
| *P*-value | 0.80 | 0.33 | 0.55 | 0.66 | 0.09 | 0.15 |
| Resources (*n* = 10) | | | | | | |
| M_1_ or M_DELTA_ | 23.30 | 26.00 | 26.30 | -2.40 | 2.20 | 5.20 |
| Mean ± SD | 23.50 ± 11.27 | 26.00 ± 1.70 | 25.50 ± 3.74 | -1.50 ± 3.86 | 3.50 ± 10.77 | 6.00 ± 6.07 |
| *P*-value | 0.98 | 0.71 | 0.30 | 0.25 | 0.01 | 0.61 |
| Mixed (*n* = 13) | | | | | | |
| M_1_ or M_DELTA_ | 53.92 | 20.62 | 25.54 | 0.46 | 9.85 | 6.23 |
| Mean ± SD | 55.00 ± 8.50 | 20.00 ± 2.84 | 26.00 ± 1.81 | 1.00 ± 3.41 | 8.00 ± 9.98 | 7.00 ± 8.34 |
| *P*-value | 0.39 | 0.03 | 0.51 | 0.94 | 0.58 | 0.30 |

Variables names are summarized in acronyms where post-traumatic stress disorder (PTSD) severity, hope-agency (HA), hope-pathways (HP), self-esteem (SE), positive mental health (PMH), well-being (WB); *P*-value is Shapiro-Wilk’s statistical normality test; M_1_ refers to the means of the variables prior to the Centre de Ressources des Blessés de l’Armée de Terre (CREBAT) course (phase 1); M_DELTA_ refers to the DELTA means of the variables measured before and after the CREBAT course
